# Ureolysis-Driven Microbially Induced Carbonate Precipitation by a Facultatively Anaerobic Thermophilic Bacterium Under High-Temperature and Anaerobic Conditions

**DOI:** 10.3390/microorganisms13051102

**Published:** 2025-05-10

**Authors:** Xiulun Shen, Sijia He, Yutaro Takaya, Tomoyoshi Yakata, Kotaro Yoshida, Hajime Kobayashi

**Affiliations:** 1 Department of Systems Innovation, School of Engineering, The University of Tokyo, Tokyo 113-8656, Japan; shen-xiulun888@g.ecc.u-tokyo.ac.jp (X.S.); he-sijia521@g.ecc.u-tokyo.ac.jp (S.H.); y-takaya@sys.t.u-tokyo.ac.jp (Y.T.); 2 Shinko Holdings Corporation, Tokyo 106-0041, Japan; t.yakata@shinko-hd.co.jp (T.Y.);; 3 Engineering for Sustainable Development of Subsurface Environments (Shinko Holdings Corporation) Social Cooperation Program, Department of Systems Innovation, School of Engineering, The University of Tokyo, Tokyo 113-8656, Japan

**Keywords:** calcium carbonate, urea hydrolysis, deep-subsurface environments, calcite, vaterite

## Abstract

Microbially induced carbonate precipitation (MICP) is the precipitation of CaCO_3_ crystals, induced by microbial metabolic activities such as ureolysis. Various applications of MICP have been proposed as innovative biocementation techniques. This study aimed to verify the feasibility of ureolysis-driven MICP applications in deep-subsurface environments (e.g., enhanced oil recovery and geological carbon sequestration). To this end, we screened sludge collected from a high-temperature anaerobic digester for facultatively anaerobic thermophilic bacteria possessing ureolytic activity. Then, we examined the ureolysis-driven MICP using a representative isolate, *Bacillus haynesii* strain SK1, under aerobic, anoxic, and strict anaerobic conditions at 30 °C, 40 °C, and 50 °C. All cultures showed ureolysis and the formation of insoluble precipitates. Fourier transform infrared spectroscopy analysis revealed precipitates comprising CaCO_3_ at 30 °C, 40 °C, and 50 °C under aerobic conditions but only at 50 °C under anoxic and strict anaerobic conditions, suggesting efficient MICP at 50 °C. Interestingly, an X-ray diffraction analysis indicated that calcium carbonate crystals that were produced under aerobic conditions were in the form of calcite, while those that were produced under anoxic and strict anaerobic conditions at 50 °C were mostly in the form of vaterite. Thus, we demonstrated ureolysis-driven MICP under high-temperature and O_2_-depletion conditions, suggesting the potential of MICP applications in deep-subsurface environments.

## 1. Introduction

Microbially induced carbonate precipitation (MICP) is the promotion of CaCO_3_ precipitation by various microbial metabolic activities, including urea hydrolysis [1,2,3,4], denitrification [5,6,7,8], sulfate reduction [9], iron reduction [10,11], methane oxidation [12], and photosynthesis [5,13]. Among them, MICP via the urea hydrolysis pathway has been intensively studied. During this process, ureolytic bacteria hydrolyze urea to ammonium and CO_2_, substantially increasing the surrounding environment’s pH and carbonate ion concentration. The bacterial cell surface and extracellular polymeric substances (e.g., extracellular polysaccharides) act as nucleation sites for mineral crystallization, facilitating CaCO_3_ crystal precipitation in the presence of sufficient calcium ions [2,14,15]. Because MICP readily creates durable carbonate depositions with a relatively low environmental impact, the applications of MICP in construction engineering and materials are diverse, including the amendment or improvement of soils and construction materials [4,11,12,16,17,18], restoration of natural sites and cultural artifacts, and water purification [10,14,19,20,21,22].

The application of MICP to control the permeability of porous media in deep-subsurface environments (i.e., porous rocks of subsurface geological strata, such as petroleum reservoirs and aquifers) has been proposed. This involves injecting fluid containing appropriate microbes and/or nutrients into the stratum to promote MICP in situ, with the resulting CaCO_3_ precipitation restricting fluid advection between neighboring pores and small aperture fractures in the rock layer, causing a net decrease in the flow rate through the stratum. Because the aqueous fluids that are used to promote MICP have relatively low viscosities, they are likely highly injectable and capable of traveling considerable horizontal and vertical distances from the injection well, resulting in CaCO_3_ crystal deposition in preferential flow paths within the stratum and thereby plugging zones with high permeability and sealing fractures. Thus, as an alternative to traditional grouting technologies, such as those using cement-based sealants [1,23,24], permeability reduction via MICP may be beneficial for improving the volumetric sweep efficiency of water injection in petroleum reservoirs for enhanced oil recovery (EOR) [25,26] and reduce the leakage risk of CO_2_ in reservoirs of geological carbon sequestration (such as aquifers) for carbon capture and storage (CCS) [15,18,27,28,29].

Laboratory-scale studies have reported that ureolysis-driven MICP effectively induces pore throat plugging and fracture sealing in porous and fractured media [18,23,30,31,32,33]. The transport behavior of a ureolytic bacterium in porous media (sandstone cores) has also been characterized [22,34,35]. Moreover, two field experiments have demonstrated reduced permeability of fractured rock by promoting ureolysis-driven MICP in relatively shallow geological formations (approximately 27 and 340 m below ground surface, respectively) [15,23]. Furthermore, a staged injection strategy was proposed for the efficient application of ureolysis-driven MICP to subsurface geological formations [36,37].

Notably, ureolysis-driven MICP has mainly been studied in surface environments under ambient conditions, almost exclusively under aerobic conditions at temperatures of 25–37 °C [38,39,40]. Many studies used *Sporosarcina pasteurii*, a mesophilic obligate aerobe that is highly capable of ureolysis-driven MICP at mesophilic temperatures of up to 40 °C, with no activity at ≥50 °C [41,42]. Moreover, *S. pasteurii* is metabolically inactive in the absence of O_2_ and incapable of MICP under anoxic conditions [43,44].

Ureolysis-driven MICP has never been demonstrated under high-temperature and O_2_-depleted conditions, such as deep-subsurface environments (e.g., petroleum reservoirs and geological carbon sequestration sites), where the temperature is high due to the geothermal gradient. Thus, we performed this proof-of-concept study to verify the feasibility of ureolysis-driven MICP applications in deep-subsurface environments. To this end, we screened facultatively anaerobic thermophilic bacteria that are capable of ureolysis and assessed ureolysis-driven MICP by a representative isolate under high-temperature and O_2_-depleted (anoxic and strict anaerobic) conditions.

## 2. Materials and Methods

### 2.1. Sample Collection

In September 2023, we collected a sludge sample from a sewage treatment plant, which operates anaerobic digestion at 50 °C, for use as the microbial source in our study. The processed sewage water with sludge was taken from a sampling port of the high-temperature anaerobic digestion reactor into sterile 50 mL tubes and stored at 4 °C until inoculation.

### 2.2. Screening and Isolation of Facultatively Anaerobic Thermophilic Bacteria with Ureolytic Properties

Christensen urea medium is a differential medium that contains phenol red, a pH indicator. Urease-producing bacteria hydrolyze the urea in the medium, yielding ammonia and increasing the alkalinity, resulting in a color change from orange to pink. We diluted the sludge sample to 1:10 with sterilized saline (0.85% [*w*/*v*] NaCl). Then, 100 µL of the diluted sample was spread onto 100 Christensen urea agar plates (1.0 g/L peptone, 1.0 g/L glucose, 20.0 g/L urea, 5.0 g/L NaCl, 2 g/L KH_2_PO_4_, 0.012 g/L phenol red, and 15.0 g/L agar) [45]; the urea and glucose were filter-sterilized [0.2 µm pore size] and added separately after autoclaving. The plates were inoculated and incubated overnight at 50 °C under aerobic conditions. Urease-positive colonies were streaked onto new plates to isolate single colonies, and this was repeated three times to ensure isolate purity. Then, the pure isolates were inoculated onto Christensen urea agar plates and incubated in an anaerobic chamber (Coy Laboratory Products, Grass Lake, MI, USA) (H_2_:CO_2_:N_2_ 10:10:80 atmosphere) to evaluate their growth and urease activity under anaerobic conditions.

### 2.3. Phylogenetic Characterization of the Isolates

Following the overnight culture of the isolates in Luria–Bertani (LB) broth (10 g/L tryptone, 10 g/L NaCl, 5 g/L yeast extract), genomic DNA was extracted using a DNeasy PowerSoil kit (Qiagen, Hilden, Germany). The extracted DNA underwent PCR using Ex Taq DNA polymerase, hot-start version (Takara Bio Inc., Kyoto, Japan), according to the manufacturer’s instructions, and the primer pair 8F (5′-AGAGTTTGATYMTGGCTCAG-3′) and 1492R (5′-CGGYTACCTTGTTACGACTT-3′) was used to target nearly the entire length of the 16S rRNA gene [46]. Thermocycling was performed as follows: initial denaturation, 5 min at 95 °C; 40 cycles of 1 min at 95 °C, 1 min at 50 °C, and 2 min at 72 °C; and a final extension for 10 min at 72 °C. The amplicons were sequenced at Macrogen Japan (Tokyo, Japan) using standard Sanger sequencing. In January 2024, the resultant 16S rRNA gene sequences were compared against sequences in the NCBI nonredundant nucleotide database (nr/nt) using blastn (https://blast.ncbi.nlm.nih.gov/Blast.cgi, accessed on 15 January 2024). We used MEGA version 12 [47] to construct a phylogenetic tree based on the Tamura–Nei model with neighbor-joining algorithms and determined the confidence levels of the nodes using 500 bootstrap replicates.

### 2.4. Carbonate Precipitation Test

A representative isolate was streaked onto an LB agar plate from a glycerol stock solution (stored at −80 °C) and incubated overnight at 30 °C. For preculture, a fresh colony was picked from the plate and inoculated into 10 mL of nutrient broth (1 g/L beef extract, 5 g/L peptone, 5 g/L NaCl, 2 g/L yeast extract), supplemented with 17.6 g/L CaCl_2_ and incubated aerobically overnight at 30 °C, 40 °C, or 50 °C (the same temperatures used in subsequent carbonate precipitation experiments) with shaking (190 rpm).

For aerobic conditions, fresh preculture was inoculated into a baffled 500 mL Erlenmeyer flask containing 50 mL of nutrient broth supplemented with 17.6 g/L CaCl_2_ and 20 g/L urea to give an initial optical density at 600 nm/cm^−1^ (OD_600_) of approximately 0.001. The cultures were incubated with shaking (190 rpm) at 30 °C, 40 °C, or 50 °C.

For anoxic and strict anaerobic conditions, the medium was supplemented with 37.7 g/L Ca(NO_3_)_2_·4H_2_O instead of CaCl_2_ as the source of calcium and nitrate (for nitrate respiration). The dissolved O_2_ was removed from the media by boiling under flowing O_2_-free Ar gas, from which the residual O_2_ was removed using a copper furnace (Sanshin, Kanagawa, Japan). For strict anaerobic conditions, 0.2% (*w*/*v*) sodium thioglycolate was added as a reducing agent and resazurin (1 mg/L) as an oxidation–reduction indicator to confirm strict anaerobic conditions [48,49]. Vial bottles (250 mL volume; Maruemu, Osaka, Japan) were used for the anoxic and strict anaerobic cultures, with inoculation being performed in the anaerobic chamber. After inoculation, the vial bottles were sealed with butyl rubber stoppers and aluminum seals. The headspaces of the cultures were replaced with O_2_-free Ar gas.

The cultures were incubated for 7 days (aerobic and anoxic conditions) or 21 days (strict anaerobic conditions). The OD_600_ of the cultures was measured using an Ultrospec 6300 Pro spectrophotometer (GE Healthcare, Chicago, IL, USA). The culture media’s pH and electrical conductivity (EC) were measured using a SevenExcellene S470-Basic pH/conductivity meter (Mettler Toledo, Greifensee, Switzerland). Experiments were performed in triplicate and included uninoculated controls.

### 2.5. Mineralogical and Morphological Analyses of the Precipitates

The precipitates that formed in the cultures were collected by centrifugation, washed three times with distilled water and 100% (*v*/*v*) ethanol, and dried at 80 °C for 24 h. Their mineralogic characteristics were analyzed using Fourier transform infrared spectroscopy (FTIR), X-ray diffraction (XRD), field emission scanning electron microscopy (SEM), and energy-dispersive X-ray spectroscopy (EDS). FTIR was performed using an Alpha II compact FTIR spectrometer (Bruker Scientific, San Jose, CA, USA) with a wavelength range of 400–4000 cm^−1^ and a resolution of 2 cm^−1^. XRD was assessed using a RINT-2100 diffractometer (Rigaku Co., Tokyo, Japan) with Cu Kα radiation (λ = 0.15406 nm) generated at 40 kV and 30 mA, with a scan rate of 2°/min for 2θ values over a wide range of Bragg angles (20° ≤ 2θ ≤ 90°). SEM and EDS were performed using a JSM-7800F scanning electron microscope (JEOL, Tokyo, Japan) with an accelerating voltage of 10 kV.

## 3. Results

### 3.1. Isolation of Facultatively Anaerobic Thermophilic Bacteria with Ureolytic Properties

**Figure 1:**
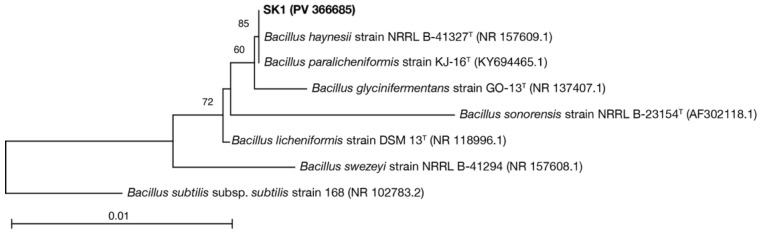
Phylogenetic relationship of *Bacillus haynesii* strain SK1 with other *Bacillus* strains. The percentage of trees in which the associated strains clustered together following 500 bootstrap replicates is indicated next to the branches. The tree is drawn to scale, with branch lengths in the same units as those of the evolutionary distances that were used to infer the phylogenetic tree. The final dataset included 1158 positions. The tree was rooted using *Bacillus subtilis* subsp. *subtilis* strain 168 (NCBI Reference Sequence: NR_102783.2).

To examine ureolysis-driven MICP under high-temperature and O_2_-depleted conditions, we screened sludge collected from a high-temperature anaerobic digester for facultatively anaerobic thermophiles possessing ureolytic activity using Christensen urea agar plates. We isolated 36 strains (SK1–SK36) that were capable of proliferation and urea hydrolysis at 50 °C under both aerobic and anaerobic conditions. A phylogenetic analysis of 16S rRNA gene sequences revealed that the isolates were highly similar to each other and closely related to bacteria of the *Bacillus licheniformis* subclade of the *B. subtilis*-*B. licheniformis* clade of the *Bacillus* genus (specifically, *B. haynesii*) (Table S1). The *B. licheniformis* subclade comprises ubiquitous Gram-positive bacteria that are widely exploited in industrial processes for the manufacturing of commercial enzymes, antibiotics, and other biochemicals [50]. These bacteria are generally thermophilic (optimum growth temperature: approximately 50 °C) and facultatively anaerobic (capable of anaerobic respiration via dissimilatory nitrate reduction to ammonium) [51,52]. Moreover, several strains in the *B. licheniformis* subclade possess urease and are capable of inducing MICP by ureolysis under aerobic conditions [53]. This suggested that bacteria belonging to the *B. licheniformis* subclade were suitable models for evaluating ureolysis-driven MICP under high-temperature and O_2_-depleted conditions. Thus, we selected a representative isolate (tentatively named *B. haynesii* strain SK1) (Figure 1) for further study.

### 3.2. Assessment of Ureolysis-Driven MICP by B. haynesii Strain SK1 Under High-Temperature and Anoxic Conditions

We assessed ureolysis-driven MICP by *B. haynesii* strain SK1 at 30 °C, 40 °C, and 50 °C under aerobic and O_2_-depleted (anoxic and strict anaerobic) conditions. For anoxic and strict anaerobic conditions, nitrate, in the form of Ca(NO_3_)_2_·4H_2_O, was used instead of CaCl_2_ to act as an electron acceptor for nitrate respiration. Dissolved O_2_ was removed from the medium by boiling, and O_2_-free Ar gas was used as the headspace gas in the cultures. Moreover, for strict anaerobic conditions, the reducing agent sodium thioglycolate was added to lower the reduction potential further.

**Figure 2:**
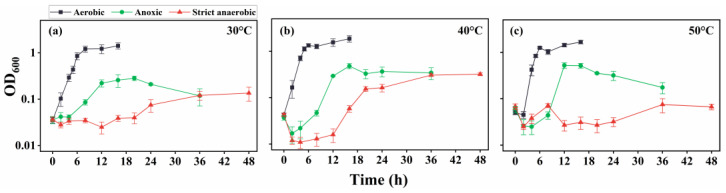
Growth curve of *B. haynesii* strain SK1 in aerobic, anoxic, and strict anaerobic conditions at 30 °C (**a**), 40 °C (**b**), and 50 °C (**c**). OD_600_, optical density at 600 nm.

The examination of SK1 in the absence of urea revealed good growth (Figure 2) under all temperature conditions (30 °C, 40 °C, and 50 °C). However, the growth rates under strict anaerobic conditions were significantly slower than those under aerobic and anoxic conditions. The doubling times for the exponential growth phases at 30 °C (Figure 2a), 40 °C (Figure 2b), and 50 °C (Figure 2c) were approximately 0.6, 0.5, and 0.3 h under aerobic conditions; 1.4, 1.1, and 0.8 h under anoxic conditions; and 4.4, 3.7, and 4.0 h under strict anaerobic conditions, respectively. Moreover, OD_600_ measurements indicated that the final yields of the cultures were generally lower under strict anaerobic conditions than those under aerobic and anoxic conditions, suggesting that SK1 metabolism was less active under strict anaerobic conditions. Notably, the reducing agent partly impaired SK1 growth, even under aerobic conditions. Moreover, other reducing agents, such as cysteine and sodium sulfide, inhibited SK1 growth more severely. This suggested that the relatively poor growth under strict anaerobic conditions was partly due to a damaging effect exerted by the reducing agent.

**Figure 3:**
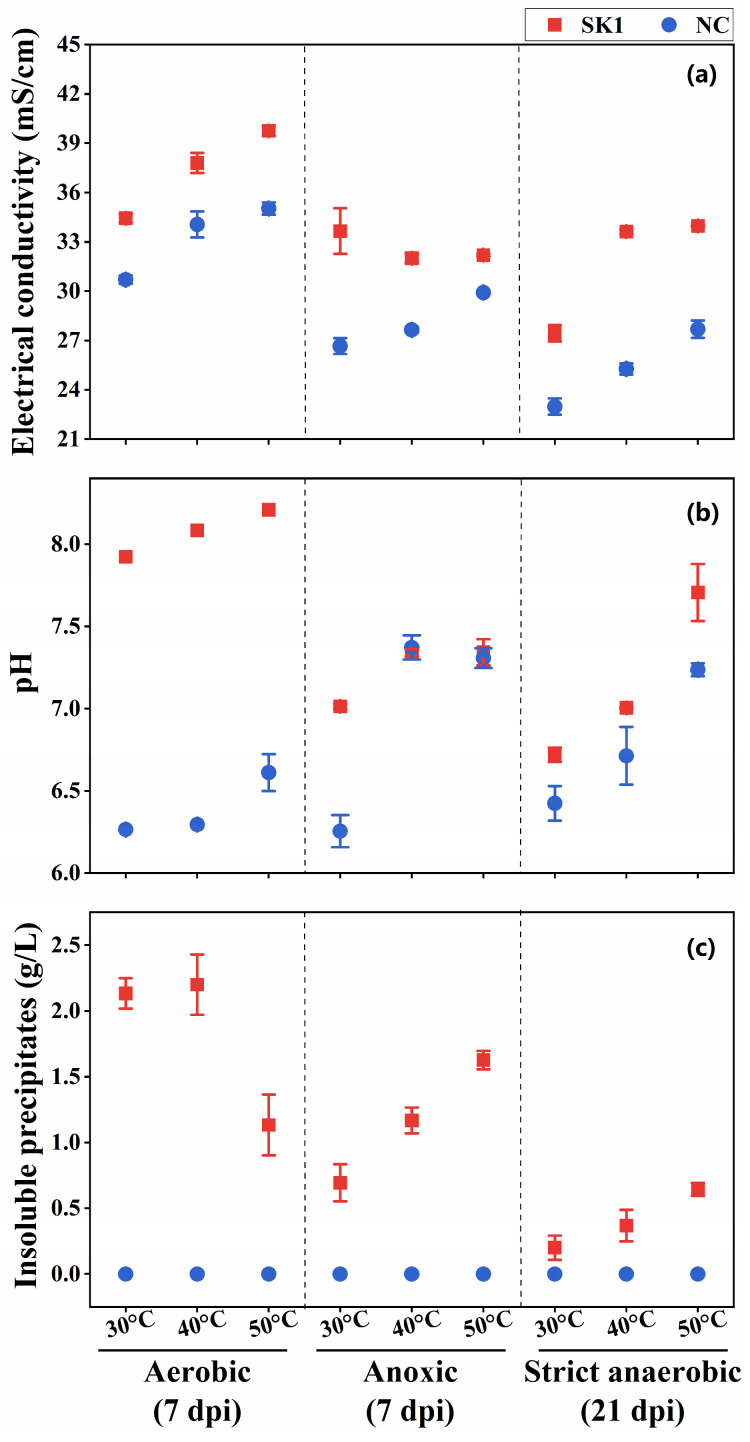
Ureolysis-driven microbially induced carbonate precipitation (MICP) of *B. haynesii* strain SK1 at 30 °C, 40 °C, and 50 °C. (**a**) Electrical conductivity, (**b**) media pH, and (**c**) precipitate formation under aerobic, anoxic, and strict anaerobic conditions. dpi, days post inoculation; NC, negative control.

We evaluated the ureolysis-driven MICP of strain SK1 under anoxic and strict anaerobic conditions at 7 days post inoculation (dpi). Under strict anaerobic conditions, however, there was no significant formation of insoluble precipitates at 7 dpi, likely due to the lower levels of metabolic activity. Thus, we re-examined the strict anaerobic cultures at 21 dpi. The EC of the inoculated culture media was generally higher than that of the uninoculated controls (Figure 3a), suggesting ureolytic activity of strain SK1 under all the conditions, resulting in increased ion concentrations. However, media alkalinization was less significant under anoxic and strict anaerobic conditions (Figure 3b), and there was no significant difference in pH between the anoxic cultures and their uninoculated controls at 40 °C and 50 °C. It is plausible that anaerobic metabolism (fermentation) by strain SK1 produced organic acids, likely preventing the alkalinization of the medium [44]. Significant formation of insoluble precipitates occurred in all cultures at 7 dpi (under aerobic and anoxic conditions) or 21 dpi (under strict anaerobic conditions). No precipitates were observed in the uninoculated controls.

**Figure 4:**
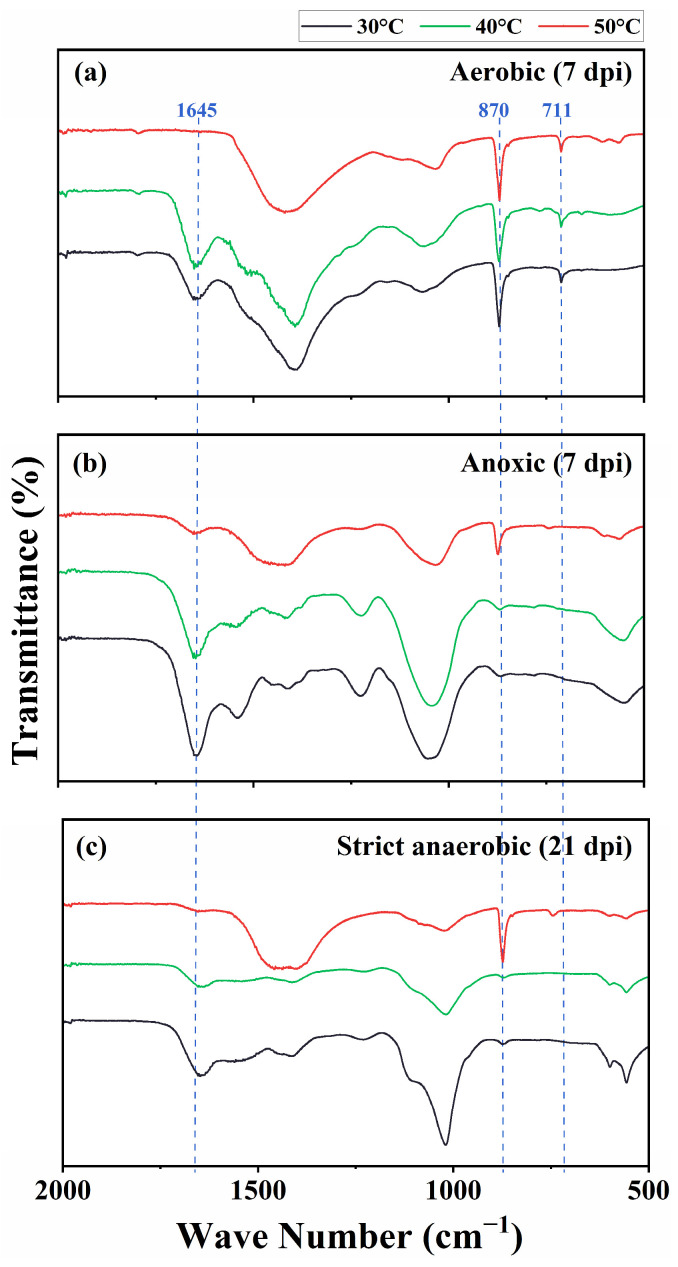
Fourier transform infrared spectroscopy (FTIR) of the insoluble precipitates produced in the cultures of *B. haynesii* strain SK1 at 30 °C, 40 °C, and 50 °C under the aerobic (**a**), anoxic (**b**), and strict anaerobic (**c**) conditions. dpi, days post inoculation.

**Figure 5:**
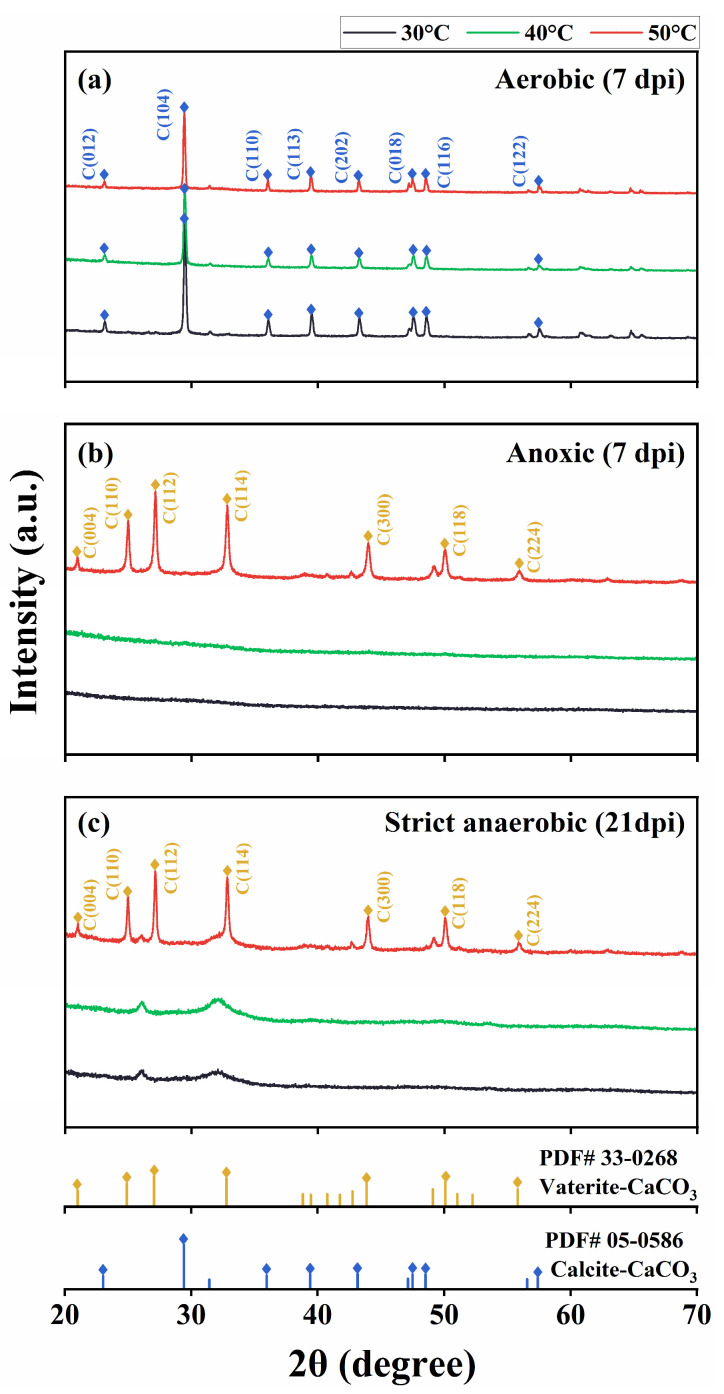
X-ray diffraction (XRD) of the insoluble precipitates produced in the cultures of *B. haynesii* strain SK1 at 30 °C, 40 °C, and 50 °C under the aerobic (**a**), anoxic (**b**), and strict anaerobic (**c**) conditions. a.u., arbitrary unit; dpi, days post inoculation.

We characterized the precipitates using FTIR, XRD, and SEM/EDS. The FTIR spectrograms showed characteristic peaks of CaCO_3_ at 870 and 711 cm^−1^ in the aerobic cultures at 30 °C, 40 °C, and 50 °C and the anoxic and strict anaerobic cultures at 50 °C. Furthermore, the precipitates from the cultures at 30 °C and 40 °C showed a peak at 1645 cm^−1^, which corresponds to the C=O stretching vibrational frequency and is indicative of protein or amide substances, likely signifying the presence of substantial biomass (Figure 4). Figure 5 shows XRD spectrograms of the precipitates. In agreement with the FTIR results, crystalline peaks were detected in the precipitates of the aerobic cultures at 30 °C, 40 °C, and 50 °C and the anoxic and strict anaerobic cultures at 50 °C. In comparison, no clear peak was detected in the precipitates of the anoxic and strict anaerobic cultures at 30 °C and 40 °C.

**Figure 6:**
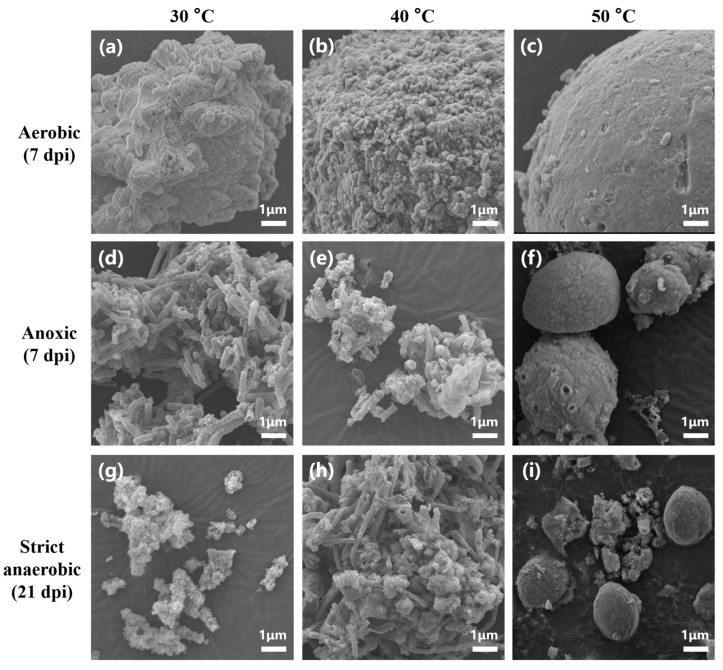
Scanning electron microscopy of the insoluble precipitates produced in the cultures of *B. haynesii* strain SK1 under aerobic, anoxic, and strict anaerobic conditions. (**a**–**c**) Mineralization under aerobic conditions at 30 °C, 40 °C, and 50 °C at 7 dpi. (**d**–**f**) Mineralization under anoxic conditions at 30 °C, 40 °C, and 50 °C at 7 dpi. (**g**–**i**) Mineralization under anaerobic conditions at 30 °C, 40 °C, and 50 °C at 21 dpi. Scale bar: 1 µm.

Interestingly, the CaCO_3_ crystals that were produced under aerobic conditions at 30 °C, 40 °C, and 50 °C were in the form of calcite, while those that were produced under anoxic and strict anaerobic conditions at 50 °C were mainly in the form of vaterite. Figure 6 and Figure S1 show SEM micrographs and EDS spectrograms, respectively, of the precipitates. Grain-like (typically globular-shaped) particles of CaCO_3_ were formed at 50 °C under aerobic, anoxic, and strict anaerobic conditions (Figure 6c,f,i), and EDS indicated that they were CaCO_3_ crystals (Figure S1c,f,i). This suggested the efficient development of CaCO_3_ crystals on the surface of bacteria at 50 °C, which likely limited cell division due to encapsulation of the individual bacterial cells within the mineral particles, resulting in the formation of grain-like particles. The mineral particles that were formed in the aerobic cultures at 50 °C were generally larger (diameter: 12.2 ± 0.3 µm) than those in the anoxic and strict anaerobic cultures at 50 °C (diameter: 6.4 ± 0.4 and 3.2 ± 0.2 µm, respectively). The smaller sizes of the mineral particles in the anoxic and strict anaerobic cultures were likely due to vaterite being less stable and more soluble than calcite. In contrast, the mineral particles in the aerobic cultures at 30 °C and 40 °C formed irregular lumps (Figure 6a,b). Rod cell-like structures (probably bacterial cells) were also observed as constituents of the lumps. This suggested that CaCO_3_ crystal development was relatively slower at 30 °C and 40 °C than at 50 °C, resulting, at least in part, in simultaneous mineral precipitation and cell proliferation. In the anoxic and strict anaerobic cultures at 30 °C and 40 °C, the precipitates appeared to be clusters of rod cell-like structures highly resembling microbial cells (Figure 6d,e,g,h). Moreover, the EDS of the rod cell-like structures indicated a high carbon content (Figure S1d,e,g,h), suggesting that those precipitates were not mineral precipitates but microbial cells. Thus, MICP was not detected in the anoxic and strict anaerobic cultures at 30 °C and 40 °C.

## 4. Discussion

We isolated a facultatively anaerobic ureolytic thermophile, *B. haynesii* strain SK1, which induced MICP via ureolysis at 50 °C under anoxic and strict anaerobic conditions. This is the first demonstration of ureolysis-driven MICP under high-temperature and O_2_-depleted conditions, suggesting the feasibility of ureolysis-driven MICP applications in deep-subsurface environments. However, the reaction kinetics and product differed depending on the culture conditions and temperatures. Under aerobic conditions, CaCO_3_ crystals precipitated at 30 °C, 40 °C, and 50 °C in the form of calcite. Under anoxic and strict anaerobic conditions, CaCO_3_ crystals only precipitated at 50 °C, mainly in the form of vaterite. Moreover, the strict anaerobic cultures produced less precipitates despite the longer incubation period (21 days) (Figure 2c), indicating that the reaction kinetic was relatively slower under strict anaerobic conditions. MICP was not observed at 30 °C and 40 °C under O_2_-depleted conditions, probably due to relatively slower crystal growth and vaterite instability at lower temperatures [54,55,56,57]. However, the mechanism determining the reaction products (calcite or vaterite) under different conditions remains unelucidated. It has been suggested that the formation and stability of CaCO_3_ crystal polymorphs can be influenced by various factors [58], including pH [59], temperature [60], ionic strength [61], inorganic [59,62,63] and organic substances [64,65,66,67,68,69], microbial metabolites [70,71] and cell surfaces [72,73], most of which can be directly or indirectly affected by the culture conditions. Generally, microorganisms modulate their metabolisms and, thus, production of metabolites in response to environmental conditions. For example, O_2_ depletion induces the nitrate respiration of strain SK1, resulting in the production of ammonium [51,52], which has been shown to promote vaterite precipitation [63]. Moreover, bacteria modify the properties of components on their outer surface (i.e., membrane proteins, lipids, surface polysaccharides, and exopolysaccharides) in response to environmental conditions [74,75,76,77]. Thus, O_2_ depletion may have altered the surface properties of *B. haynesii* strain SK1 to favor vaterite crystal formation. Furthermore, under anoxic and strict anaerobic conditions, the alkalinization of the medium was not significant (Figure 3b), probably because of organic acid production due to anaerobic metabolic activity [78]. In addition, culture conditions other than the O_2_ status may also affect crystal precipitation. For instance, the anoxic and strict anaerobic cultures were tightly sealed by butyl rubber stoppers (i.e., closed systems), so the CO_2_ produced by the bacteria accumulated within the cultures and may have affected the media properties, such as the pH and dissolved CO_2_ and carbonate concentrations. Thus, future studies are needed to thoroughly understand the effects of environmental conditions on microbial physiology and ureolysis-driven MICP. Particularly, identifying the limiting factors (such as competitive reactions) [79,80] or additives facilitating crystal deposition (such as surfactants, amino acids, proteins, polymers, alcohols, and microorganisms) [65,66,67,69,81,82,83] will be useful not only to further understand the reaction mechanism but also to establish a methodology to enhance ureolysis-driven MICP for actual applications. There has been a wealth of such studies on ureolysis-driven MICP under ambient (moderate)-temperature and aerobic conditions [84,85,86,87,88,89]. On the other hand, this study is the first report of ureolysis-driven MICP under high-temperature and O_2_-depleted conditions, providing a basis to warrant future studies for its optimization and applications in deep-subsurface environments.

In real-world subsurface applications, such as EOR and CCS, the target environments (e.g., petroleum reservoirs and aquifers) are generally anaerobic. However, the injection fluids containing microbes, calcium, electron acceptors, and urea will have been prepared in and injected from surface facilities. Thus, the injected slug is likely to carry dissolved O_2_. The injected microbes are expected to induce ureolysis-driven MICP mainly within the injected slug, the in situ condition of which will likely be under an O_2_ concentration gradient from (micro)oxic to anoxic or strict anaerobic conditions. Thus, facultatively anaerobic thermophiles, such as bacteria in the *B. licheniformis* subclade, are preferable to strict anaerobes for real-world applications, because they can easily be propagated under aerobic (surface) conditions and remain metabolically active under O_2_-depleted conditions. In future studies, however, it is necessary to examine ureolysis-driven MICP under conditions that are more relevant to the real-world deep-subsurface environments, such as under high pressures and wider ranges of temperatures and O_2_ gradients, in media that are closer to actual groundwaters and injection fluids, and within solid porous materials (i.e., sandstones and carbonate rocks). In addition, natural environments generally harbor complex microbiotas comprising diverse microorganisms with various metabolic abilities. Under such conditions, components such as organic acids and CO_2_ produced by a bacterium are readily consumed by other microorganisms, such as indigenous acetoclastic or chemolithotrophic anaerobes [90,91]. Furthermore, new ureolytic bacteria with higher tolerance to such challenging conditions of deep-subsurface environments will be necessary for future research. This study used a sludge sample as a microbial source specifically to screen facultatively anaerobic ureolytic thermophiles. However, the environment within the high-temperature anaerobic digester significantly differs from the deep-subsurface environments in terms of physicochemical conditions. The strain SK1 is not able to grow at 60 °C or above, making examinations at higher temperatures impossible in this study. Moreover, because the rate of ureolysis-driven MICP by *B. haynesii* strain SK1 was relatively slow under strict anaerobic conditions, further screening for facultatively anaerobic thermophiles with higher urease activity is necessary.

## 5. Conclusions

This study demonstrated ureolysis-driven MICP by *B. haynesii* strain SK1, a facultatively anaerobic thermophile, under anoxic and strict anaerobic conditions at 50 °C. Furthermore, it also revealed that the reaction product at 50 °C (vaterite) differed from the reaction product formed under aerobic conditions (calcite). This is the first report of ureolysis-driven MICP under high-temperature and O_2_-depleted conditions, suggesting the possibility of MICP applications in deep-subsurface environments. However, investigations of MICP under more realistic conditions mimicking subsurface environments, such as in the presence of complex indigenous microbiota instead of a pure culture, and further screening of facultatively anaerobic thermophiles with more potent ureolysis properties are warranted.

## Data Availability

The partial 16S rRNA gene sequences of isolates SK1~SK36 have been deposited in the GenBank database under accession numbers PV366685-PV366720.

## Supplementary Materials

The following supporting information can be downloaded at https://www.mdpi.com/article/10.3390/microorganisms13051102/s1: Supplemental Figure S1 (Energy-dispersive X-ray spectroscopy of the mineralization products under aerobic, anoxic, and strict anaerobic conditions); Table S1 (Phylogenetic analysis of the isolated strains based on 16S rRNA gene sequences).

## Abbreviations

The following abbreviations are used in this manuscript:

CCS	Carbon capture and storage
dpi	Days post inoculation
EC	Electrical conductivity
EDS	Energy-dispersive X-ray spectroscopy
EOR	Enhanced oil recovery
FTIR	Fourier transform infrared spectroscopy
LB	Luria–Bertani
MICP	Microbially induced carbonate precipitation
NC	Negative control
SEM	Scanning electron microscopy
XRD	X-ray diffraction

## References

[1] Boquet E., Boronat A., Ramos-Cormenzana A. (1973). Production of calcite (calcium carbonate) crystals by soil bacteria is a general phenomenon. Nature.

[2] Phillips A.J., Gerlach R., Lauchnor E., Mitchell A.C., Cunningham A.B., Spangler L. (2013). Engineered applications of ureolytic biomineralization: A review. Biofouling.

[3] Zhu T.T., Dittrich M. (2016). Carbonate precipitation through microbial activities in natural environment, and their potential in biotechnology: A Review. Front. Bioeng. Biotechnol..

[4] Gat D., Ronen Z., Tsesarsky M. (2016). Soil bacteria population dynamics following stimulation for ureolytic microbial-induced CaCO_3_ precipitation. Environ. Sci. Technol..

[5] Ersan Y., de Belie N., Boon N. (2015). Microbially induced CaCO_3_ precipitation through denitrification: An optimization study in minimal nutrient environment. Biochem. Eng. J..

[6] Martin D., Dodds K., Butler I.B., Ngwenya B.T. (2013). Carbonate precipitation under pressure for bioengineering in the anaerobic subsurface via denitrification. Environ. Sci. Technol..

[7] Feng X., Guo H.G., Feng X.Y., Yin Y.F., Li Z.G., Huang Z.X., Urynowicz M. (2024). Denitrification induced calcium carbonate precipitation by indigenous microorganisms in coal seam and its application potential in CO_2_ geological storage. Fuel.

[8] Gao Y.Q., Wang L.Y., He J., Ren J., Gao Y.F. (2022). Denitrification-based MICP for cementation of soil: Treatment process and mechanical performance. Acta Geotech..

[9] Ganendra G., De Muynck W., Ho A., Arvaniti E.C., Hosseinkhani B., Ramos J.A., Rahier H., Boon N. (2014). Formate oxidation-driven calcium carbonate precipitation by methylocystis parvus OBBP. Appl. Environ. Microb..

[10] Taharia M., Dey D., Das K., Sukul U., Chen J.S., Banerjee P., Dey G., Sharma R.K., Lin P.Y., Chen C.Y. (2024). Microbial induced carbonate precipitation for remediation of heavy metals, ions and radioactive elements: A comprehensive exploration of prospective applications in water and soil treatment. Ecotox. Environ. Safe.

[11] Seifan M., Berenjian A. (2019). Microbially induced calcium carbonate precipitation: A widespread phenomenon in the biological world. Appl. Microbiol. Biot..

[12] Fouladi A.S., Arulrajah A., Chu J., Horpibulsuk S. (2023). Application of microbially induced calcite precipitation (MICP) technology in construction materials: A comprehensive review of waste stream contributions. Constr. Build. Mater..

[13] Liu Y., Ali A., Su J.F., Li K., Hu R.Z., Wang Z. (2023). Microbial-induced calcium carbonate precipitation: Influencing factors, nucleation pathways, and application in waste water remediation. Sci. Total Environ..

[14] Bai H., Liu D., Zheng W.L., Ma L.Y., Yang S.S., Cao J.P., Lu X.L., Wang H.M., Mehta N. (2021). Microbially-induced calcium carbonate precipitation by a halophilic ureolytic bacterium and its potential for remediation of heavy metal-contaminated saline environments. Int. Biodeter. Biodegr..

[15] Phillips A.J., Cunningham A.B., Gerlach R., Hiebert R., Hwang C., Lomans B.P., Westrich J., Mantilla C., Kirksey J., Esposito R. (2016). Fracture sealing with microbially-induced calcium carbonate precipitation: A field study. Environ. Sci. Technol..

[16] Phillips A.J., Troyer E., Hiebert R., Kirkland C., Gerlach R., Cunningham A.B., Spangler L., Kirksey J., Rowe W., Esposito R. (2018). Enhancing wellbore cement integrity with microbially induced calcite precipitation (MICP): A field scale demonstration. J. Pet. Sci. Eng..

[17] Celia M.A., Bachu S., Nordbotten J.M., Bandilla K.W. (2015). Status of CO_2_ storage in deep saline aquifers with emphasis on modeling approaches and practical simulations. Water Resour. Res..

[18] Phillips A.J., Lauchnor E., Eldring J., Esposito R., Mitchell A.C., Gerlach R., Cunningham A.B., Spangler L.H. (2013). Potential CO_2_ leakage reduction through biofilm-induced calcium carbonate precipitation. Environ. Sci. Technol..

[19] Rajasekar A., Wilkinson S., Moy C.K.S. (2021). MICP as a potential sustainable technique to treat or entrap contaminants in the natural environment: A review. Environ. Sci. Ecotechnol..

[20] Hu X.M., Liu J.D., Feng Y., Zhao Y.Y., Wang X.W., Liu W.H., Zhang M., Liu Y. (2023). Application of urease-producing microbial community in seawater to dust suppression in desert. Environ. Res..

[21] He Z.F., Xu Y.T., Yang X.L., Shi J.F., Wang X., Jin Z.Z., Zhang D.Y., Pan X.L. (2022). Passivation of heavy metals in copper-nickel tailings by in-situ bio-mineralization: A pilot trial and mechanistic analysis. Sci. Total Environ..

[22] Lauchnor E.G., Schultz L.N., Bugni S., Mitchell A.C., Cunningham A.B., Gerlach R. (2013). Bacterially induced calcium carbonate precipitation and strontium coprecipitation in a porous media flow system. Environ. Sci. Technol..

[23] Cuthbert M.O., McMillan L.A., Handley-Sidhu S., Riley M.S., Tobler D.J., Phoenix V.R. (2013). A field and modeling study of fractured rock permeability reduction using microbially induced calcite precipitation. Environ. Sci. Technol..

[24] Soon N.W., Lee L.M., Khun T.C., Ling H.S. (2014). Factors affecting improvement in engineering properties of residual soil through microbial-induced calcite precipitation. J. Geotech. Geoenviron. Eng..

[25] Wu J., Wang X.B., Wang H.F., Zeng R.J. (2017). Microbially induced calcium carbonate precipitation driven by ureolysis to enhance oil recovery. RSC Adv..

[26] Xia S.X., Davletshin A., Song W. (2023). Enhanced oil recovery through microbially induced calcium carbonate precipitation. Energy Fuels.

[27] Bhukya P.K., Adla N., Arnepalli D.N. (2024). Numerical optimisation of microbially induced calcite precipitation (MICP) injection strategies for sealing the aquifer’s leakage paths for CO_2_ geosequestration application. Adv. Water Res..

[28] Kirkland C.M., Akyel A., Hiebert R., McCloskey J., Kirksey J., Cunningham A.B., Gerlach R., Spangler L., Phillips A.J. (2021). Ureolysis-induced calcium carbonate precipitation (UICP) in the presence of CO_2_-affected brine: A field demonstration. Int. J. Greenh. Gas Control.

[29] Mitchell A.C., Phillips A., Schultz L., Parks S., Spangler L., Cunningham A.B., Gerlach R. (2013). Microbial CaCO_3_ mineral formation and stability in an experimentally simulated high pressure saline aquifer with supercritical CO_2_. Int. J. Greenh. Gas Control.

[30] Ferris F., Stehmeier L., Kantzas A., Mourits F. (1996). Bacteriogenic mineral plugging. J. Can. Petrol. Technol..

[31] Sham E., Mantle M., Mitchell J., Tobler D., Phoenix V., Johns M. (2013). Monitoring bacterially induced calcite precipitation in porous media using magnetic resonance imaging and flow measurements. J. Contam. Hydrol..

[32] Tobler D.J., Maclachlan E., Phoenix V.R. (2012). Microbially mediated plugging of porous media and the impact of differing injection strategies. Ecol. Eng..

[33] van Paassen L.A., Ghose R., van der Linden T.J., van der Star W.R., van Loosdrecht M.C. (2010). Quantifying biomediated ground improvement by ureolysis: Large-scale biogrout experiment. J. Geotech. Geoenviron. Eng..

[34] Tobler D.J., Cuthbert M.O., Phoenix V.R. (2014). Transport of sporosarcina pasteurii in sandstone and its significance for subsurface engineering technologies. Appl. Geochem..

[35] Zambare N.M., Lauchnor E.G., Gerlach R. (2019). Controlling the distribution of microbially precipitated calcium carbonate in radial flow environments. Environ. Sci. Technol..

[36] Fujita Y., Taylor J.L., Gresham T.L.T., Delwiche M.E., Colwell F.S., McLing T.L., Petzke L.M., Smith R.W. (2008). Stimulation of microbial urea hydrolysis in groundwater to enhance calcite precipitation. Environ. Sci. Technol..

[37] Hommel J., Lauchnor E., Gerlach R., Cunningham A.B., Ebigbo A., Helmig R., Class H. (2016). Investigating the influence of the initial biomass distribution and injection strategies on biofilm-mediated calcite precipitation in porous mMedia. Transp. Porous Media.

[38] Li M.D., Wen K.J., Li Y., Zhu L.P. (2018). Impact of Oxygen availability on microbially induced calcite precipitation (MICP) Treatment. Geomicrobiol. J..

[39] Kim G., Kim J., Youn H. (2018). Effect of temperature, pH, and reaction duration on microbially induced calcite precipitation. Appl. Sci..

[40] Dong Y.R., Gao Z.Q., Wang D., Di J.Z., Guo X.Y., Yang Z.H., Li Y., Wang Y.H., Wang Y.S. (2023). Optimization of growth conditions and biological cementation effect of Sporosarcina pasteurii. Constr. Build. Mater..

[41] Yi H.H., Zheng T.W., Jia Z.R., Su T., Wang C.G. (2021). Study on the influencing factors and mechanism of calcium carbonate precipitation induced by urease bacteria. J. Cryst. Growth.

[42] Omoregie A.I., Khoshdelnezamiha G., Senian N., Ong D.E.L., Nissom P.M. (2017). Experimental optimisation of various cultural conditions on urease activity for isolated Sporosarcina pasteurii strains and evaluation of their biocement potentials. Ecol. Eng..

[43] Martin D., Dodds K., Ngwenya B.T., Butler I.B., Elphick S.C. (2012). Inhibition of Sporosarcina pasteurii under anoxic conditions: Implications for subsurface carbonate precipitation and remediation via ureolysis. Environ. Sci. Technol..

[44] Jain S., Arnepalli D.N. (2019). Biochemically induced carbonate precipitation in aerobic and anaerobic environments by Sporosarcina pasteurii. Geomicrobiol. J..

[45] Anitha V. (2018). *Bacillus cereus* KLUVAA mediated biocement production using hard water and urea. Chem. Biochem. Eng. Q..

[46] Feng X., He S.J., Sato T., Kondo T., Uema K., Sato K., Kobayashi H. (2023). Enrichment of hydrogen-oxidizing bacteria using a hybrid biological-inorganic system. J. Biosci. Bioeng..

[47] Kumar S., Stecher G., Suleski M., Sanderford M., Sharma S., Tamura K. (2024). MEGA12: Molecular evolutionary genetic analysis version 12 for adaptive and green computing. Mol. Biol. Evol..

[48] Bala-Amutha K., Murugesan A.G. (2013). Biohydrogen production using corn stalk employing *Bacillus licheniformis* MSU AGM 2 strain. Renew. Energy.

[49] Javaheri M., Jenneman G.E., McInerney M.J., Knapp R.M. (1985). Anaerobic production of a biosurfactant by *Bacillus licheniformis* JF-2. Appl. Environ. Microb..

[50] Muras A., Romero M., Mayer C., Otero A. (2021). Biotechnological applications of *Bacillus licheniformis*. Crit. Rev. Biotechnol..

[51] Li J., Du C., Liu Z., Li X. (2022). Electrochemical studies of microbiologically influenced corrosion of x80 steel by nitrate-reducing *Bacillus licheniformis* under anaerobic conditions. J. Mater. Sci. Technol..

[52] Zhao N., Yu T., Yan F.J. (2023). Probiotic role and application of thermophilic Bacillus as novel food materials. Trends Food Sci. Technol..

[53] Helmi F.M., Elmitwalli H.R., Elnagdy S.M., El-Hagrassy A.F. (2016). Calcium carbonate precipitation induced by ureolytic bacteria *Bacillus licheniformis*. Ecol. Eng..

[54] Kralj D., Brečević L., Nielsen A.E. (1994). Vaterite growth and dissolution in aqueous solution II. kinetics of dissolution. J. Cryst. Growth.

[55] Kralj D., Brečević L., Nielsen A.E. (1990). Vaterite growth and dissolution in aqueous solution I. kinetics of crystal growth. J. Cryst. Growth.

[56] Kralj D., Brečević L., Kontrec J. (1997). Vaterite growth and dissolution in aqueous solution III. kinetics of transformation. J. Cryst. Growth.

[57] Bergwerff L., van Paassen L.A. (2021). Review and recalculation of growth and nucleation kinetics for calcite, vaterite and amorphous calcium carbonate. Crystals.

[58] Trushina D.B., Bukreeva T.V., Kovalchuk M.V., Antipina M.N. (2014). CaCO_3_ vaterite microparticles for biomedical and personal care applications. Mater. Sci. Eng. C-Mater. Biol. Appl..

[59] Rodriguez-Navarro C., Jimenez-Lopez C., Rodriguez-Navarro A., Gonzalez-Muñoz M.T., Rodriguez-Gallego M. (2007). Bacterially mediated mineralization of vaterite. Geochim. Cosmochim. Acta.

[60] Chen J., Xiang L. (2009). Controllable synthesis of calcium carbonate polymorphs at different temperatures. Powder Technol..

[61] Spanos N., Koutsoukos P.G. (1998). The transformation of vaterite to calcite: Effect of the conditions of the solutions in contact with the mineral phase. J. Cryst. Growth.

[62] Gehrke N., Cölfen H., Pinna N., Antonietti M., Nassif N. (2005). Superstructures of calcium carbonate crystals by oriented attachment. Cryst. Growth Des..

[63] Hu Q.N., Zhang J.M., Teng H., Becker U. (2012). Growth process and crystallographic properties of ammonia-induced vaterite. Am. Mineral..

[64] Lv C., Tang C.S., Zhang J.Z., Liu H., Pan X.H. (2025). Dissolution and recrystallization behavior of microbially induced calcium carbonate: Influencing factors, kinetics, and cementation effect. Can. Geotech. J..

[65] Manoli F., Kanakis J., Malkaj P., Dalas E. (2002). The effect of aminoacids on the crystal growth of calcium carbonate. J. Cryst. Growth.

[66] Malkaj P., Kanakis J., Dalas E. (2004). The effect of leucine on the crystal growth of calcium carbonate. J. Cryst. Growth.

[67] Wang X.Q., Wu C.M., Tao K., Zhao K., Wang J.Q., Xu H., Xia D.H., Shan H.H., Lu J.R. (2010). Influence of ovalbumin on CaCO_3_ precipitation during in vitro biomineralization. J. Phys. Chem. B.

[68] Yao C.L., Xu W.H., Ding A.M., Zhu J.M. (2009). Sucrose/bovine serum albumin mediated biomimetic crystallization of calcium carbonate. J. Chem. Sci..

[69] Liu Y., Cui Y.J., Mao H.Y., Guo R. (2012). Calcium carbonate crystallization in the presence of casein. Cryst. Growth Des..

[70] Kawaguchi T., Decho A.W. (2002). A laboratory investigation of cyanobacterial extracellular polymeric secretions (EPS) in influencing CaCO_3_ polymorphism. J. Cryst. Growth.

[71] Tourney J., Ngwenya B.T. (2009). Bacterial extracellular polymeric substances (EPS) mediate CaCO_3_ morphology and polymorphism. Chem. Geol..

[72] Ghosh T., Bhaduri S., Montemagno C., Kumar A. (2019). *Sporosarcina pasteurii* can form nanoscale calcium carbonate crystals on cell surface. PLoS ONE.

[73] Szczes A., Czemierska M., Jarosz-Wilkolazka A., Magierek E., Chibowski E., Holysz L. (2018). Extracellular polymeric substance of *Rhodococcus opacus* bacteria effects on calcium carbonate formation. Physicochem. Probl. Miner. Process..

[74] Nielsen A.K., Breüner A., Krzystanek M., Andersen J.T., Poulsen T.A., Olsen P.B., Mijakovic I., Rasmussen M.D. (2010). Global transcriptional analysis of *Bacillus licheniformis* reveals an overlap between heat shock and iron limitation stimulon. J. Mol. Microbiol. Biotechnol..

[75] Ardissone S., Kobayashi H., Kambara K., Rummel C., Noel K.D., Walker G.C., Broughton W.J., Deakin W.J. (2011). Role of BacA in lipopolysaccharide synthesis, peptide transport, and nodulation by *Rhizobium* sp. strain NGR234. J. Bacteriol..

[76] Voigt B., Schroeter R., Jürgen B., Albrecht D., Evers S., Bongaerts J., Maurer K.H., Schweder T., Hecker M. (2013). The response of *Bacillus licheniformis* to heat and ethanol stress and the role of the SigB regulon. Proteomics.

[77] Dong Z.X., Chen Z.X., Wang H.B., Tian K.M., Jin P., Liu X.G., McHunu N.P., Permaul K., Singh S., Niu D.D. (2017). Tandem mass tag-based quantitative proteomics analyses reveal the response of *Bacillus licheniformis* to high growth temperatures. Ann. Microbiol..

[78] Shariati P., Mitchell W.J., Boyd A., Priest F.G. (1995). Anaerobic metabolism in *Bacillus licheniformis* NCIB 6346. Microbiology.

[79] Li L., Qian C.X., Cheng L., Wang R.X. (2010). A laboratory investigation of microbe-inducing CdCO_3_ precipitate treatment in Cd^2+^ contaminated soil. J. Soils Sediments.

[80] Johnstone E.V., Hofmann S., Cherkouk A., Schmidt M. (2016). Study of the interaction of Eu^3+^ with microbiologically induced c Carbonate precipitates using TRLFS. Environ. Sci. Technol..

[81] Dupont L., Portemer F., Figlarz M. (1997). Synthesis and study of a well crystallized CaCO_3_ vaterite showing a new habitus. J. Mater. Chem..

[82] Qi L.M., Li J., Ma J.M. (2002). Biomimetic morphogenesis of calcium carbonate in mixed solutions of surfactants and double-hydrophilic block copolymers. Adv. Mater..

[83] Zhao Y.Y., Du W., Sun L.M., Yu L., Jiao J.J., Wang R. (2013). Facile synthesis of calcium carbonate with an absolutely pure crystal form using 1-butyl-3-methylimidazolium dodecyl sulfate as the modifier. Colloid Polym. Sci..

[84] Zhao Y.X., Peng L.G., Feng Z.Y., Lu Z.M. (2021). Optimization of microbial induced carbonate precipitation treatment process to improve recycled fine aggregate. Clean. Mater..

[85] Fu T., Saracho A.C., Haigh S.K. (2023). Microbially induced carbonate precipitation (MICP) for soil strengthening: A comprehensive review. Biogeotechnics.

[86] Zhuo X., Fan L., Hu D., Zhu H. (2022). Multifactor optimization of MICP base on BP model. J. Phys. Conf. Ser..

[87] Pan Z.K., Cao S.D. (2024). Optimization of culture medium to improve bio-cementation effect based on response surface method. Sci. Rep..

[88] Dagliya M., Satyam N., Garg A. (2023). Optimization of growth medium for microbially induced calcium carbonate precipitation (MICP) treatment of desert sand. J. Arid Land.

[89] Lv C., Tang C.S., Zhu C., Li W.Q., Chen T.Y., Zhao L., Pan X.H. (2022). Environmental Dependence of microbially induced calcium carbonate crystal precipitations: Experimental evidence and insights. J. Geotech. Geoenviron. Eng..

[90] Kobayashi H., Goto A., Feng X., Uruma K., Momoi Y., Watanabe S., Sato K., Zhang Y., Horne R.N., Shibuya T. (2023). Long-term microbial DNA-based monitoring of the mature sarukawa oil field in Japan. SPE Reserv. Eval. Eng..

[91] Kobayashi H., Endo K., Sakata S., Mayumi D., Kawaguchi H., Ikarashi M., Miyagawa Y., Maeda H., Sato K. (2012). Phylogenetic diversity of microbial communities associated with the crude-oil, large-insoluble-particle and formation-water components of the reservoir fluid from a non-flooded high-temperature petroleum reservoir. J. Biosci. Bioeng..

