# Supplementary material for: Ureolysis-Driven Microbially Induced Carbonate Precipitation by a Facultatively Anaerobic Thermophilic Bacterium Under High-Temperature and Anaerobic Conditions
Source: Microorganisms. 2025 May 10;13(5):1102. doi: 10.3390/microorganisms13051102 (PMC12113697; doi:10.3390/microorganisms13051102)
Supplement: Supplementary file 1 [file microorganisms-13-01102-s001.zip › Supplementary Materials.pdf]

Supplementary Materials

Supplemental Figure.

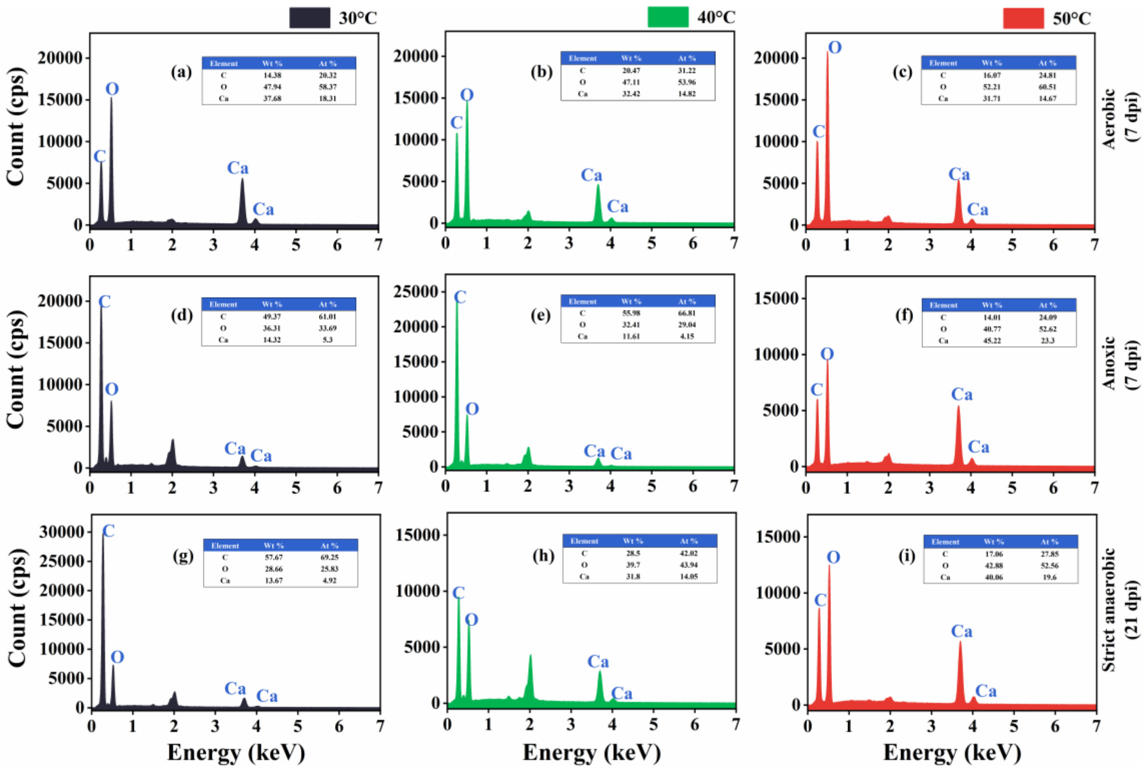

Figure S1. Energy-dispersive X-ray spectroscopy of the insoluble precipitates produced in the cultures of *B. haynesii* strain SK1 under aerobic, anoxic, and strict anaerobic conditions. (a–c) Mineralization under the aerobic condition at 30°C, 40°C, and 50°C at 7 dpi. (d–f) Mineralization under the anoxic condition at 30°C, 40°C, and 50°C at 7 dpi. (g-i) Mineralization under the anaerobic condition at 30°C, 40°C, and 50°C at 21 dpi. Scale bar: 1 μm.

Supplemental Table.

Table S1. Phylogenetic analysis of the isolated strains based on 16S rRNA gene sequences.

Strain	Top hit*	Identity (%)
SK1	*Bacillus haynesii* strain NRRL B-41327 (NR 157609.1)	100
SK2	*Bacillus haynesii* strain NRRL B-41327 (NR 157609.1)	99.927
SK3	*Bacillus haynesii* strain NRRL B-41327 (NR 157609.1)	100
SK4	*Bacillus haynesii* strain NRRL B-41327 (NR 157609.1)	99.284
SK5	*Bacillus haynesii* strain NRRL B-41327 (NR 157609.1)	99.506
SK6	*Bacillus haynesii* strain NRRL B-41327 (NR 157609.1)	98.997
SK7	*Bacillus haynesii* strain NRRL B-41327 (NR 157609.1)	99.571
SK8	*Bacillus haynesii* strain NRRL B-41327 (NR 157609.1)	99.635
SK9	*Bacillus haynesii* strain NRRL B-41327 (NR 157609.1)	99.582
SK10	*Bacillus haynesii* strain NRRL B-41327 (NR 157609.1)	98.611
SK11	*Bacillus haynesii* strain NRRL B-41327 (NR 157609.1)	96.671
SK12	*Bacillus haynesii* strain NRRL B-41327 (NR 157609.1)	99.569
SK13	*Bacillus haynesii* strain NRRL B-41327 (NR 157609.1)	99.57
SK14	*Bacillus haynesii* strain NRRL B-41327 (NR 157609.1)	99.638
SK15	*Bacillus haynesii* strain NRRL B-41327 (NR 157609.1)	99.708
SK16	*Bacillus haynesii* strain NRRL B-41327 (NR 157609.1)	99.712
SK17	*Bacillus haynesii* strain NRRL B-41327 (NR 157609.1)	99.351
SK18	*Bacillus haynesii* strain NRRL B-41327 (NR 157609.1)	99.503
SK19	*Bacillus haynesii* strain NRRL B-41327 (NR 157609.1)	99.071
SK20	*Bacillus haynesii* strain NRRL B-41327 (NR 157609.1)	99.153
SK21	*Bacillus haynesii* strain NRRL B-41327 (NR 157609.1)	99.713
SK22	*Bacillus haynesii* strain NRRL B-41327 (NR 157609.1)	99.646
SK23	*Bacillus haynesii* strain NRRL B-41327 (NR 157609.1)	99.718
SK24	*Bacillus haynesii* strain NRRL B-41327 (NR 157609.1)	99.283
SK25	*Bacillus haynesii* strain NRRL B-41327 (NR 157609.1)	99.642
SK26	*Bacillus haynesii* strain NRRL B-41327 (NR 157609.1)	99.569
SK27	*Bacillus haynesii* strain NRRL B-41327 (NR 157609.1)	94.77
SK28	*Bacillus haynesii* strain NRRL B-41327 (NR 157609.1)	99.581
SK29	*Bacillus haynesii* strain NRRL B-41327 (NR 157609.1)	99.86
SK30	*Bacillus haynesii* strain NRRL B-41327 (NR 157609.1)	98.634
SK31	*Bacillus haynesii* strain NRRL B-41327 (NR 157609.1)	99.511
SK32	*Bacillus haynesii* strain NRRL B-41327 (NR 157609.1)	99.647
SK33	*Bacillus haynesii* strain NRRL B-41327 (NR 157609.1)	99.65
SK34	*Bacillus haynesii* strain NRRL B-41327 (NR 157609.1)	99.443

SK35	*Bacillus haynesii* strain NRRL B-41327 (NR 157609.1)	99.71
SK36	*Bacillus haynesii* strain NRRL B-41327 (NR 157609.1)	96.108

*Top hits by BLAST-N search in the NCBI database were shown with the acceesion numbers (in parentheses).
